# Dairy Intake and Type 2 Diabetes Mellitus in Iranian Older Adults: Insights From the Baseline Phase of the Birjand Longitudinal Aging Study

**DOI:** 10.1002/edm2.70196

**Published:** 2026-04-09

**Authors:** Mahboubeh Darabi, Amirmohammad Tajik, Mehraneh Movahedi Aliabadi, Zeinab Shakeri, Maryam Mohammadi, Hossein Fakhrzadeh, Mitra Moudi, Masoumeh Khorashadizadeh, Mehdi Varmaghani, Farshad Sharifi

**Affiliations:** ^1^ Department of Management Sciences and Health Economy, Faculty of Health Mashhad University of Medical Sciences Mashhad Iran; ^2^ School of Pharmacy Mashhad University of Medical Sciences Mashhad Iran; ^3^ Health Policy Research Center, Institute of Health Shiraz University of Medical Sciences Shiraz Iran; ^4^ Orthopedics Research Center Mashhad University of Medical Sciences Mashhad Iran; ^5^ Department of Health Education and Health Promotion, School of Health Mashhad University of Medical Sciences Mashhad Iran; ^6^ Social Determinants of Health Research Center Mashhad University of Medical Sciences Mashhad Iran; ^7^ Elderly Health Research Center, Endocrinology and Metabolism Population Sciences Institute Tehran University of Medical Sciences Tehran Iran; ^8^ Geriatric Health Research Center Birjand University of Medical Sciences Birjand Iran; ^9^ School of Public Health Birjand University of Medical Sciences Birjand Iran; ^10^ Department of Gerontology, School of Rehabilitation Tehran University of Medical Sciences Tehran Iran

**Keywords:** cheese, dairy products, health services for aged, type 2 diabetes mellitus, yogurt

## Abstract

**Background:**

Type 2 diabetes mellitus (T2DM) is a growing public health concern among older adults worldwide including in Iran. Despite extensive research on the metabolic effects of dairy products, their association with T2DM remains inconsistent, particularly in non‐Western populations. This study examined the relationship between dairy consumption including specific types, and T2DM among older adults in eastern Iran.

**Methods:**

This cross‐sectional study utilised baseline data from the Birjand Longitudinal Aging Study (BLAS), which included community‐dwelling adults aged 60 years and older. Dietary intake was assessed using structured questionnaires, and participants were categorised into tertiles based on dairy consumption (low, moderate, high). T2DM status was determined by FBS ≥ 126 mg/dL or a previous physician diagnosis. Logistic regression models estimated the odds ratios (ORs) and 95% confidence intervals (CIs) for T2DM across different levels of dairy intake, adjusting for potential confounders.

**Results:**

Data from 1348 participants were analysed, with an overall T2DM prevalence of 27.15%. Individuals in the highest tertiles of yogurt and cheese intake had significantly increased odds of T2DM (OR = 1.454, 95% CI = 1.08–2.20, *p* = 0.08; OR = 1.44, 95% CI = 1.04–2.00, *p* = 0.029). No significant association was found between milk consumption and T2DM risk. Total dairy consumption showed no significant association with T2DM in the fully adjusted model (OR = 1.42, 95% CI = 1.00–2.403, *p* = 0.05).

**Conclusions:**

This study reveals a complex association between dairy intake and the risk of T2DM in Iran, in which high consumption of yogurt and cheese was paradoxically associated with increased odds of disease. This finding may be explained by the high fat content of these products, residual confounding from unmeasured dietary patterns, or biological pathways related to diabetes pathology and gut microbiota modulation that were not captured in our analysis.

## Graphical abstract

Among 1348 Iranian adults aged ≥ 60 years in the BLAS baseline, total dairy intake showed no clear association with prevalent T2DM (adjusted OR: 1.42, *p* = 0.05). Higher yogurt (OR: 1.48–1.54) and cheese (OR: 1.44) intakes were linked to greater odds, whereas milk was not.

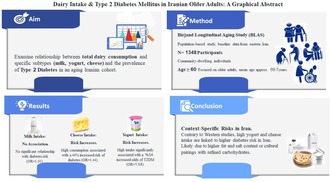

## 1 Introduction

Type 2 diabetes mellitus (T2DM) is a multifactorial disease characterised by insulin resistance and impaired glucose regulation. T2DM, in particular, is one of the fastest‐growing non‐communicable diseases globally, with profound implications for aging populations [1]. Age is a significant risk factor in the development of T2DM, with the risk increasing as individuals age [2].

According to the latest estimates from the International Diabetes Federation (IDF) for 2025, approximately 589 million people (aged 20–79 years) worldwide are living with diabetes, representing about 11.1% of the global adult population (roughly one in every nine adults). A significant majority of these individuals (over 90%) have T2DM. By 2050, it is estimated that 1 in 8 adults, approximately 853 million, will be living with diabetes, marking a 46% increase. It is worth noting that over 4 in 5 adults (81%) with diabetes live in low‐ and middle‐income countries [3]. Iran mirrors this global trend, with national data indicating a diabetes prevalence of approximately 10%, disproportionately affecting older adults and women [4, 5]. The most recent data from the IDF indicates that approximately 5.5 million people (aged 20–79 years) are living with diabetes in Iran in 2024 [3, 4, 5]. Although Iran is not currently among the countries with the largest populations of older adults, due to a sharp decline in the population growth rate, it is expected to become one of the countries with the largest populations of older adults in the near future [6]. Therefore, it is crucial to investigate the factors associated with the incidence of T2DM among older Iranian adults, especially those that play a significant role such as diet.

Although aging is an important factor, environmental and lifestyle factors, particularly diet, are critical in shaping diabetes risk [7]. Among dietary components, dairy products have garnered considerable attention due to their complex nutrient profile, which includes calcium, vitamin D, whey proteins, and bioactive compounds [8, 9]. Several mechanisms have been proposed to explain the potential antidiabetic properties of dairy [10, 11]. For instance, whey protein has been shown to stimulate postprandial insulin secretion, reduce appetite via incretin pathways (e.g., GLP‐1), and delay gastric emptying [12, 13]. Fermented dairy products, such as yogurt, are rich in probiotics that may modulate gut microbiota, leading to reduced endotoxemia and improved insulin sensitivity [14, 15]. Additionally, dairy‐derived saturated fats may have a different metabolic profile compared to those from animal meat, particularly in terms of cardiometabolic outcomes [16, 17]. However, the relationship between dairy intake and the risk of T2DM remains inconclusive [18]. Studies from different regions of the world yield conflicting results. Western populations generally show an inverse association between dairy consumption and T2DM risk, particularly with yogurt and low‐fat dairy products. In contrast, findings from Asian and Middle Eastern countries have been less consistent, with some suggesting a potential increased risk, possibly due to factors like lactose intolerance, dietary habits, or types of dairy products consumed [9, 19, 20, 21, 22, 23, 24].

In Iran, dairy consumption is both nutritionally and culturally significant but differs markedly from Western norms. Full‐fat yogurt and cheese are commonly consumed in high quantities, often with added salt, sugar, or preservatives. Low‐fat or unflavoured alternatives, which are commonly studied in other regions, are less prevalent [25, 26]. Therefore, the generalizability of international findings to Iranian populations, particularly older adults, is limited. Moreover, few studies in Iran have examined the association between specific dairy subtypes (e.g., yogurt, milk and cheese) and diabetes risk, and even fewer have focused on older adults, despite their increased vulnerability to diet‐related chronic conditions.

To address this knowledge gap, the present study investigates the association between dairy consumption and T2DM among older adults using data from the Birjand Longitudinal Aging Study (BLAS). BLAS is a population‐based cohort of community‐dwelling individuals aged 60 years or older in eastern Iran [27]. This study not only explores overall dairy intake but also examines the potential differential effects of specific dairy products, namely milk, yogurt and cheese, on diabetes prevalence, whereas accounting for key confounders such as obesity, physical activity and hypertension. This approach seeks to provide culturally relevant evidence in the field of nutritional epidemiology, particularly regarding the relationship between dairy consumption and T2DM risk in aging populations in Iran, where findings often contradict those from Western studies and cannot be directly generalised to non‐Western populations.

## 2 Methods

### 2.1 Study Design and Population

This cross‐sectional study analysed data from the first wave of the Birjand Longitudinal Aging Study (BLAS). The BLAS is a prospective cohort study involving older adults aged 60 years and above, residing in the city of Birjand, located in eastern Iran. Data for the first phase of BLAS, which involved 1420 participants, were collected between October 2018 and April 2019. Inclusion criteria required participants to be aged 60 or older, residing in Birjand, and able to attend the research center, complete the questionnaire, undergo physical examination and provide laboratory data. Exclusion criteria included bedridden individuals, those with severe cognitive impairments, or those unable to communicate effectively. Additionally, older adults who declined to participate were excluded from the study [27].

Eight trained researchers conducted face‐to‐face interviews with the participants, with one researcher responsible for data collection in each domain. Data were gathered using an online software tool called Digit, which ensures the validation of values and prevents incomplete form submissions [27]. Additional details regarding the study design and data collection process can be found elsewhere [28, 29, 30, 31].

### 2.2 Assessment of Baseline Characteristics

The collected data included demographic information such as age, sex, marital status, educational level and occupation. Height and weight were measured without shoes and with minimal clothing, using a precision of 0.5 cm and 0.1 kg, respectively. BMI was calculated by dividing weight (in kilograms) by the square of height (in meters) and categorised into two groups: non‐obese (BMI < 30 kg/m^2^) and obese (BMI ≥ 30 kg/m^2^). Waist circumference was measured using a nonelastic tape held horizontally just above the iliac crest in a standing position after a normal exhalation. Waist circumference was categorised into two groups: non‐abdominal obesity (male: < 102 cm, female: < 88 cm) and abdominal obesity (male: ≥ 102 cm, female: ≥ 88 cm). Physical activity was assessed using the Longitudinal Aging Study Amsterdam Physical Activity Questionnaire (LAPAQ), which evaluates physical activity levels at work and during leisure time. The LAPAQ is an 18‐item tool that assesses the frequency and duration of various activities over the past 2 weeks. The results were converted to metabolic equivalents of tasks (METs) to represent physical activity levels [32]. Physical activity levels were categorised as inactive (< 600 MET‐minutes/week) and active (≥ 600 MET‐minutes/week).

### 2.3 Diabetes and Hypertension Diagnosis

Chronic diseases, including hypertension and T2DM, were defined by self‐report or informant‐reported clinician diagnosis, in combination with two‐time blood pressure measurements (mean blood pressure ≥ 140/90 mmHg) and fasting blood sugar (FBS) ≥ 126 mg/dL [33]. Participants who reported a medical diagnosis of hypertension or diabetes and were using blood pressure or glucose‐lowering medications were also included. FBS was measured using the glucose oxidase enzymatic method with colorimetric detection [27].

### 2.4 Assessment of Dairy Consumption

To assess dairy product intake, we utilised the standardised Iranian adaptation of the Food Frequency Questionnaire (FFQ)‐14 [34]. Participants were asked about the frequency of their consumption (daily, weekly, monthly, yearly) of five dairy items: milk, yogurt, doogh (an Iranian yogurt drink), curd and cheese. The survey also inquired about the daily quantity of dairy products consumed (in servings). Participants were categorised into three levels of dairy intake: less than 1 serving daily (first tertile), 1–2 servings daily (second tertile) and ≥ 3 servings daily (third tertile).

### 2.5 Statistical Analysis

After data collection and verification, all statistical analyses were performed using Stata version 16 (StataCorp LP, College Station, Texas, USA). Continuous variables were described using the mean and standard deviation (SD), whereas categorical variables were summarised as frequencies (percentages). The Chi‐square test was used to examine relationships between qualitative variables and diabetes mellitus. This test assessed the significance of differences in frequency distributions across groups. The independent *t*‐test was used to compare the means of quantitative variables between diabetic and non‐diabetic individuals. Normality was assessed using the Kolmogorov–Smirnov test before conducting *t*‐tests. To examine the relationship between dairy consumption and diabetes, a logistic regression model was applied, adjusting for potential confounders such as age, sex, education, marital status, occupation, physical activity level, BMI, waist circumference, hypertension, high‐fat dairy intake, wholegrain bread and number of servings of red meat per day. Results are presented as odds ratios (OR) with 95% confidence intervals (CIs), and a *p*‐value less than 0.05 was considered statistically significant.

## 3 Results

### 3.1 Baseline Characteristics of Participants

A total of 1420 subjects were initially enrolled in the first phase of the BLAS. After excluding participants with incomplete data, a cohort of 1348 older adults aged 60 years and above, with a mean age of 69.73 ± 7.53 years, was analysed in this study. The demographic and clinical characteristics of the participants are summarised in Table 1. Among the study cohort, 48.29% were male and 51.71% were female. The largest proportion of participants (56.16%) was aged 60–69 years, whereas 31.31% were in the 70–79 age range, and 12.54% were aged 80 years or older. Regarding educational attainment, a significant proportion of participants (44.96%) were classified as illiterate. Examination of marital status revealed that 18.18% of participants were single, whereas the majority were married. In terms of employment, a substantial majority (82.42%) were either homemakers or retirees, with a smaller proportion employed (12.61%) or unemployed (4.97%). Regarding medical conditions, 27.15% of participants were diagnosed with T2DM, and 56.53% had hypertension. The distribution of obesity types revealed nearly equal proportions of individuals with and without abdominal obesity (49.41% and 50.59%, respectively). Additionally, a majority of participants (78.86%) were classified as non‐obese based on BMI, whereas 21.14% were categorised as obese according to standard criteria.

**TABLE 1:** Basic characteristics of study participants.

Characteristics	Diabetes		Total (*N* = 1348)	*p* *
	No (*N* = 982, 72.85%)	Yes (*N* = 366, 27.15%)		
Age, years (*M* ± SD)	69.88 ± 7.66	69.33 ± 7.39	69.73 ± 7.53	0.235
Age group (*N*, %)				
60–69	539, 71.20	218, 28.80	757, 56.16	0.100
70–79	309, 73.22	113, 26.78	422, 31.31	
> 80	134, 79.29	35, 20.71	169, 12.54	
Sex (*N*, %)				
Female	484, 69.44	213, 30.56	697, 51.71	**0.004**
Male	498, 76.50	153, 23.50	651, 48.29	
Education, years (*M* ± SD)	4.14 ± 4.96	4.94 ± 5.23	4.36 ± 5.04	**0.009**
Education level (*N*, %)				
Illiterate	460, 75.91	146, 24.09	606, 44.96	0.124
Lower than a diploma	356, 71.06	145, 28.94	501, 37.17	
Diploma	93, 69.92	40, 30.08	133, 9.87	
Academic	73, 67.59	35, 32.41	108, 8.01	
Marital status (*N*, %)				
Single	177, 72.24	68, 27.76	245, 18.19	0.798
Married	805, 73.05	297, 26.95	1102, 81.81	
Job status (*N*, %)				
Retired	359, 71.23	145, 28.77	504, 37.39	**0.001**
Employed	140, 82.35	30, 17.65	170, 12.61	
Housekeeper	425, 70.02	182, 29.98	607, 45.03	
Unemployed	58, 86.57	9, 2.46	67, 4.97	
Hypertension (*N*, %)				
No	489, 83.45	97, 16.55	586, 43.47	**< 0.001**
Yes	493, 64.70	269, 35.30	762, 56.53	
BMI (*M* ± SD)	25.85 ± 5.21	27.93 ± 5.28	26.42 ± 5.31	**< 0.001**
BMI (*N*, %)				
Not obese	801, 75.35	262, 24.65	1063, 78.86	**< 0.001**
Obese	181, 63.51	104, 36.49	285, 21.14	
METs minute weekly (*M* ± SD)	1665.53 ± 2501.08	1782.29 ± 3121.14	1697.23 ± 2682.93	0.477
Physical activity (*N*, %)				
Inactive	473, 71.56	188, 28.44	661, 49.07	0.276
Active	509, 74.20	177, 25.80	686, 50.93	
Waist circumference (*M* ± SD)	93.67 ± 12.06	99.45 ± 9.99	95.24 ± 11.82	**< 0.001**
Abdominal obesity (*N*, %)				
No	531, 79.73	135, 20.27	666, 49.41	**< 0.001**
Yes	451, 66.13	231, 33.87	682, 50.59	
Dairy consumption (*N*, %)				
First tertile	328, 72.89	122, 27.11	450, 33.38	0.835
Second tertile	331, 73.72	118, 26.28	449, 33.31	
Third tertile	323, 71.94	126, 28.06	449, 33.31	
Wholegrain bread consumption				
No	277, 28.21	100, 27.32	377, 27.97	0.747
Yes	705, 71.79	266, 72.68	971, 72.03	
Number of serving red meat per day				
≤ 1 serving	409, 41.65	156, 42.62	565, 41.91	0.475
2–4 servings	560, 57.03	202, 55.19	762, 56.53	
≥ 5 servings	13, 1.32	8, 2.19	21, 1.56	

* Significant *p*‐values are presented in bold.

### 3.2 The Association Between Baseline Characteristics and Diabetes

Table 1 shows a significant correlation between diabetes prevalence and several demographic factors. Diabetes prevalence was significantly higher in females (30.56%) compared to males (23.50%) (*p* = 0.004). The average years of education among individuals with diabetes (4.94 ± 5.23) was significantly greater than that of those without diabetes (4.14 ± 4.96) (*p* = 0.009). Occupational status was also significantly associated with diabetes prevalence (*p* = 0.001). The highest prevalence of diabetes was observed among homemakers (29.98%) and retirees (28.77%), whereas the lowest prevalence was seen in employed individuals (17.65%) and the unemployed (2.46%).

A robust and significant correlation between anthropometric measures and diabetes prevalence was found. The mean BMI for individuals with diabetes (27.93 ± 5.28) was significantly higher than for those without diabetes (25.85 ± 5.21) (*p* < 0.001). Additionally, the prevalence of diabetes was significantly higher among obese individuals (36.49%) compared to non‐obese individuals (24.65%) (*p* < 0.001). Waist circumference, a measure of central obesity, was strongly associated with diabetes. The mean waist circumference for individuals with diabetes (99.45 ± 9.99 cm) was significantly greater than for those without diabetes (93.67 ± 12.06 cm) (*p* < 0.001). The prevalence of diabetes in individuals with abdominal obesity (33.87%) was significantly higher than in those without abdominal obesity (20.27%) (*p* < 0.001).

Hypertension was also significantly associated with diabetes. The prevalence of diabetes in individuals with hypertension (35.30%) was higher than in those with normal blood pressure (16.55%). In contrast, physical activity and MET‐minutes/week did not show a significant association with diabetes status (*p* = 0.276 and *p* = 0.477, respectively). Dairy consumption also did not reveal a significant association with diabetes (*p* = 0.835).

### 3.3 The Association Between Dairy Consumption and Diabetes

Multiple logistic regression analysis was used to explore the relationship between dairy consumption frequency and the likelihood of developing diabetes. Four sequential regression models were implemented, each adding a new set of control variables to adjust for confounding factors. Dairy consumption was categorised into three groups based on weekly intake: once a week, twice a week, and three times a week, with the once‐a‐week group as the reference. The analysis outcomes are shown in Table 2. The crude model examined the association between dairy consumption frequency and diabetes prevalence before adjusting for any confounding variables. The results revealed no statistically significant association between dairy consumption and diabetes in the crude model or in models one and two. However, in models three and four, where additional variables were adjusted, the odds ratio for high dairy consumption (third tertile) increased and approached statistical significance. These findings suggest that high dairy consumption may be linked to an elevated risk of diabetes, although the association did not reach significance at *p* < 0.05 (model 3: OR = 1.36, 95% CI = 0.98–1.89, *p* = 0.06; final model: OR = 1.42, 95% CI = 1.00–2.03, *p* = 0.05).

**TABLE 2:** Adjusted odds ratios and 95% confidence intervals for total dairy consumption categories.

Models	Total dairy consumption category	OR (95% CI)	Std. err.	*Z*	*p*
Crude	First (Ref.)	1			
	Second	0.95 (0.71–1.28)	0.14	−0.28	0.77
	Third	1.04 (0.78–1.40)	0.15	0.32	0.75
Model 1^a^	First (Ref.)	1			
	Second	0.99 (0.73–1.33)	0.15	−0.06	0.95
	Third	1.16 (0.86–1.58)	0.17	1.02	0.31
Model 2^b^	First (Ref.)	1		1	
	Second	0.93 (0.68–1.25)	0.14	−0.47	0.64
	Third	1.03 (0.75–1.41)	0.16	0.21	0.83
Model 3^c^	First (Ref.)	1		1	
	Second	1.00 (0.74–1.37)	0.15	0.06	0.95
	Third	1.36 (0.98–1.89)	0.22	1.84	0.06
Model 4^d^	First (Ref.)	1		1	
	Second	1.06 (0.76–1.47)	0.18	0.35	0.72
	Third	1.42 (1.00–2.03)	0.26	1.95	0.05

^a^ Model 1: Adjusted for age and sex.

^b^ Model 2: Additional adjustment for age, sex, education years, marital status, job status and METs minutes weekly.

^c^ Model 3: Additional adjustment for age, sex, education years, marital status, job status, METs minutes weekly, BMI and waist circumference.

^d^ Model 4: Additional adjustment for age, sex, education years, marital status, job status, METs minutes weekly, BMI, waist circumference, hypertension, high‐fat dairy, wholegrain bread and number of serving red meat per day.

Further analysis focused on specific dairy products. Table 3 presents the results for yogurt consumption. In model 4, consumption of yogurt in the second and third tertiles was significantly associated with higher odds of diabetes compared to the lowest tertile (OR = 1.48, CI = 1.09–2.02, *p* = 0.012; and OR = 1.54, CI = 1.08–2.20, *p* = 0.018). This indicates that moderate and high yogurt consumers (i.e., second and third tertiles) were 48% and 54% more likely to develop diabetes, respectively, compared to the lowest consumption group (i.e., first tertile).

**TABLE 3:** Adjusted odds ratios and 95% confidence intervals for tertile yogurt consumption categories.

Models	Yogurt consumption category	OR (95% CI)	Std. err.	*Z*	*p* *
Crude	First (Ref.)	1			
	Second	1.44 (1.09–1.92)	0.21	2.55	**0.010**
	Third	1.57 (1.13–2.17)	0.26	2.72	**0.006**
Model 1^a^	First (Ref.)	1			
	Second	1.52 (1.14–2.02)	0.22	2.86	**0.004**
	Third	1.70 (1.22–2.36)	0.28	3.16	**0.002**
Model 2^b^	First (Ref.)	1		1	
	Second	1.44 (1.08–1.93)	0.21	2.50	**0.012**
	Third	1.49 (1.06–2.10)	0.25	2.34	**0.019**
Model 3^c^	First (Ref.)	1		1	
	Second	1.42 (1.04–1.92)	0.21	2.28	**0.020**
	Third	1.47 (1.03–2.08)	0.26	2.15	**0.030**
Model 4^d^	First (Ref.)	1		1	
	Second	1.48 (1.09–2.02)	0.23	2.50	**0.012**
	Third	1.54 (1.08–2.20)	0.28	2.37	**0.018**

* Significant *p*‐values are presented in bold.

^a^ Model 1: Adjusted for age and sex.

^b^ Model 2: Additional adjustment for age, sex, education years, marital status, job status and METs minutes weekly.

^c^ Model 3: Additional adjustment for age, sex, education years, marital status, job status, METs minutes weekly, BMI and waist circumference.

^d^ Model 4: Additional adjustment for age, sex, education years, marital status, job status, METs minutes weekly, BMI, waist circumference, hypertension, high‐fat dairy, wholegrain bread and number of serving red meat per day.

For cheese consumption, as shown in Table 4, a statistically significant association with an increased risk of diabetes was observed only in the third tertile of cheese consumption (highest consumption). After adjusting for confounding factors in model 4, individuals in the third tertile of cheese consumption were 39% more likely to develop diabetes compared to the first tertile (OR = 1.44, 95% CI = 1.04–2.00, *p* = 0.029). No such association was found with moderate cheese consumption (second tertile).

**TABLE 4:** Adjusted odds ratios and 95% confidence intervals for tertile cheese consumption categories.

Models	Cheese consumption category	OR (95% CI)	Std. err.	*Z*	*p* *
Crude	First (Ref.)	1			
	Second	1.27 (0.95–1.69)	0.18	1.65	0.090
	Third	1.54 (1.15–2.07)	0.23	2.89	**0.004**
Model 1^a^	First (Ref.)	1			
	Second	1.26 (0.94–1.68)	0.18	1.58	0.110
	Third	1.55 (1.15–2.08)	0.23	2.91	**0.004**
Model 2^b^	First (Ref.)	1		1	
	Second	1.17 (0.87–1.57)	0.17	1.09	0.270
	Third	1.42 (1.04–1.92)	0.21	2.27	**0.023**
Model 3^c^	First (Ref.)	1		1	
	Second	1.17 (0.86–1.57)	0.17	1.03	0.300
	Third	1.38 (1.01–1.88)	0.21	2.06	**0.040**
Model 4^d^	First (Ref.)	1		1	
	Second	1.19 (0.87–1.64)	0.19	1.10	0.271
	Third	1.44 (1.04–2.00)	0.24	2.19	**0.029**

* Significant *p*‐values are presented in bold.

^a^ Model 1: Adjusted for age and sex.

^b^ Model 2: Additional adjustment for age, sex, education years, marital status, job status and METs minutes weekly.

^c^ Model 3: Additional adjustment for age, sex, education years, marital status, job status, METs minutes weekly, BMI and waist circumference.

^d^ Model 4: Additional adjustment for age, sex, education years, marital status, job status, METs minutes weekly, BMI, waist circumference, hypertension, high‐fat dairy, wholegrain bread and number of serving red meat per day.

Table 5 shows that milk consumption was not significantly associated with diabetes risk. The odds ratios for the second and third tertiles of milk consumption were close to 1 in all models (OR = 1.09, CI = 0.81–1.48; and OR = 1.01, CI = 0.71–1.43), with *p*‐values well above the significance threshold (*p* = 0.57 and *p* = 0.96), suggesting that milk intake does not significantly impact diabetes risk.

**TABLE 5:** Adjusted odds ratios and 95% confidence intervals for tertile milk consumption categories.

Models	Milk consumption category	OR (95% CI)	Std. err.	*Z*	*p*
Crude	First (Ref.)	1			
	Second	1.04 (0.79–1.38)	0.14	0.31	0.75
	Third	1.00 (0.73–1.37)	0.16	0.02	0.98
Model 1^a^	First (Ref.)	1			
	Second	1.09 (0.82–1.45)	0.15	0.66	0.51
	Third	1.07 (0.77–1.48)	0.17	0.42	0.67
Model 2^b^	First (Ref.)	1		1	
	Second	1.10 (0.82–1.46)	0.16	0.65	0.51
	Third	1.00 (0.72–1.40)	0.16	0.05	0.96
Model 3^c^	First (Ref.)	1		1	
	Second	1.08 (0.80–1.45)	0.16	0.54	0.58
	Third	1.00 (0.71–1.40)	0.17	0.01	0.98
Model 4^d^	First (Ref.)	1		1	
	Second	1.09 (0.81–1.48)	0.17	0.57	0.57
	Third	1.01 (0.71–1.43)	0.18	0.05	0.96

^a^ Model 1: Adjusted for age and sex.

^b^ Model 2: Additional adjustment for age, sex, education years, marital status, job status and METs minutes weekly.

^c^ Model 3: Additional adjustment for age, sex, education years, marital status, job status, METs minutes weekly, BMI and waist circumference.

^d^ Model 4: Additional adjustment for age, sex, education years, marital status, job status, METs minutes weekly, BMI, waist circumference, hypertension, high‐fat dairy, wholegrain bread and number of serving red meat per day.

## 4 Discussion

This cross‐sectional study of older Iranian adults reveals a nuanced association between dairy intake and T2DM. We found that overall dairy consumption had a near‐significant relationship with diabetes; however, high intakes of specific dairy subtypes, particularly yogurt and cheese, were associated with a higher risk of T2DM. These findings are intriguing given the mixed evidence globally on dairy and metabolic health, and they highlight the importance of considering local dietary patterns and product types.

In Western cohorts, greater dairy consumption (especially yogurt and low‐fat dairy) is often linked to lower incidence of T2DM [35, 36, 37]. For example, a prospective study in Spanish older adults at high cardiovascular risk (PREDIMED cohort) reported that those with the highest yogurt intake had significantly reduced diabetes risk [36]. Likewise, meta‐analyses of predominantly Western cohorts indicate that total dairy intake, particularly low‐fat dairy and fermented products, tends to have a modest protective effect against T2DM [35, 38]. A pooled analysis of three U.S. cohorts estimated a nearly 17% reduction in T2DM incidence per daily serving of yogurt [37]. These benefits have been attributed to dairy's unique nutrients and fermentation byproducts. Calcium and vitamin D may improve insulin action, whereas whey proteins can stimulate insulin secretion and satiety hormones. Additionally, probiotic cultures in yogurt may enhance gut microbiota and metabolic health [39, 40]. Such mechanisms could explain the generally inverse association between yogurt consumption and T2DM observed in Western populations.

In contrast, our results align with a growing body of research from Asian and Middle Eastern settings where dairy's effects appear less favourable or even detrimental [41, 42, 43]. A comprehensive meta‐analysis found that higher dairy intake was associated with an increased risk of T2DM in Asian and Australian populations, even though no such association was observed in North America or Europe [42]. Recent findings from Iran support our observations: a cross‐sectional analysis from the Persian cohort reported that consuming milk and cheese was positively associated with prevalent T2DM [43]. The authors attributed this discrepancy from Western studies to traditional Iranian dietary habits, notably a generally low milk intake and higher consumption of refined carbohydrates (e.g., bread) paired with dairy, as well as differences in the types of dairy products consumed locally [41]. Similarly, an extensive, multi‐country study (PURE) that included populations from the Middle East and South Asia found that only whole‐fat dairy (not low‐fat) was associated with a lower prevalence of metabolic syndrome and diabetes [23]. Notably, a recent cohort study in Sweden reported that extremely high consumption of non‐fermented milk and cheese was associated with an elevated risk of T2DM, whereas fermented dairy products (yogurt, sour milk) and even butter showed modest protective associations. Those authors observed that an additional 100 g/day of milk increased the risk of diabetes by approximately 4%, whereas a further 100 g of fermented dairy reduced the risk by nearly 3%. Interestingly, the detrimental association of cheese in that study was evident primarily in men [44]. Taken together, global data underscore that the relationship between dairy and diabetes is complex and may vary by dairy subtype, preparation method, intake level, and population characteristics [45].

Our finding that high yogurt intake was linked to greater diabetes risk runs counter to much of the Western literature. One explanation might lie in how yogurt is prepared and consumed in Iran. Traditional Iranian diets often include yogurt in forms that differ from the plain, probiotic‐rich yogurts studied in Western cohorts. For instance, yogurt in Iran may be served with added salt (e.g., doogh, a salted yogurt drink) or sugar, or used as a condiment with large meals rich in refined carbohydrates. These practices could counteract the potential benefits of yogurt [46]. Salt content is an important consideration, as high sodium intake can contribute to hypertension and insulin resistance, potentially worsening metabolic risk despite yogurt's probiotic qualities. Similarly, any added sugars or sweetened yogurt desserts would increase glycemic load. Moreover, the fermentation process and bacterial strains in locally produced yogurts might differ from those in commercial probiotic yogurts elsewhere. Emerging evidence suggests that the specific microbes and fermentation by‐products in yogurt can influence metabolic outcomes [39]. If Iranian yogurts have fewer live cultures or different strains, their impact on gut microbiota and insulin sensitivity may be diminished. It is also plausible that reverse causation or residual confounding played a role: older adults with diabetes or obesity might gravitate toward consuming more yogurt as a substitute for other desserts or to aid digestion, thereby creating a spurious positive association. Although we adjusted for major confounders, unmeasured factors could still influence the observed link.

Cheese intake in our study was also positively associated with diabetes risk, a finding that mirrors some reports from Western populations but contradicts others. In the aforementioned U.S. cohorts, a higher consumption of cheese was associated with an increased incidence of T2DM [37]. Researchers speculate that cheese's high saturated fat content may attenuate insulin sensitivity and promote weight gain, thereby increasing the risk of diabetes. Cheese is calorically dense and often high in salt; therefore, frequent cheese consumption may contribute to higher energy intake and increased blood pressure, two factors associated with metabolic syndrome and diabetes [45]. Additionally, in Iranian culture, cheese is commonly eaten with bread (typically refined white flatbread). This pairing may acutely increase postprandial glucose and insulin demands and, over time, could exacerbate insulin resistance. On the other hand, not all studies agree on the benefits of cheese. Some European analyses have found neutral or even inverse associations when consumed in moderation [38]. It has been proposed that minerals like calcium and bioactive peptides from cheese protein may offset some of the adverse effects of dairy fat by improving pancreatic β‐cell function or reducing fat absorption [40]. The net impact of cheese likely depends on the balance between these beneficial nutrients and the harmful loads of saturated fat and salt. Our results suggest that in this Iranian cohort, the latter influences may predominate at high intake levels.

Interestingly, total dairy intake was not significantly related to T2DM in our analysis, reflecting a net null effect of mixed dairy sources. This outcome aligns with several extensive prospective studies that observed no overall association when all dairy products were combined [38]. It appears that the benefits of certain dairy types can be offset by the risks of others, yielding a neutral aggregate effect [45]. Genetic evidence also suggests that there is no strong causal effect of dairy on diabetes: a recent Mendelian randomization review found no clear link between genetically predicted milk consumption and T2DM risk [47]. Such findings reinforce that it is the type and quality of dairy, rather than total quantity alone, that matters. In our study, milk consumption was relatively low and showed no significant association with T2DM. This may be due in part to the population's genetics. Lactase persistence is relatively uncommon in Iran, with estimates indicating that less than 10% of some ethnic groups carry lactase‐persistence alleles [48]. Consequently, many older adults avoid drinking large amounts of milk and instead rely on yogurt and cheese. The low prevalence of lactase persistence and the generally low per capita milk intake in Iran suggest that the potential benefits of milk observed in other populations may not be fully realised in this context.

The divergent health effects of dairy products can be partially explained by their composition and processing. Dairy foods are a complex matrix of nutrients and bioactive compounds. On one hand, they provide calcium, magnesium and potassium (which may improve insulin signalling and blood pressure control) and contain specific fatty acids that might be beneficial [40]. Fermented dairy also supplies probiotics and fermentation metabolites that can enhance gut health and potentially reduce systemic inflammation [39, 45]. Whey proteins stimulate the release of incretin hormones and insulin, thereby improving postprandial glycemic control. These factors form the biological basis for why moderate dairy intake, especially fermented, low‐fat varieties, has been considered metabolically favourable [49]. On the other hand, dairy products high in saturated fat can promote dyslipidemia and ectopic fat accumulation, leading to insulin resistance [45]. Cheese and some full‐fat yogurts also tend to be high in sodium, and frequent excess salt intake is linked to hypertension and possibly hyperinsulinemia. Additionally, consuming added sugars in flavoured dairy products or alongside sugary foods would increase the glycemic load. Processing methods, such as fermentation type and bacterial strains, may further alter effects [46]. Our findings imply that in the Iranian context, the potentially adverse aspects of yogurt and cheese processing may outweigh their inherent benefits.

Alternatively, it is possible that the increased odds of diabetes associated with yogurt consumption are not directly causal but rather reflect residual confounding from unmeasured dietary habits. Our analysis may not have fully captured the complexity of participants' overall diets. For instance, yogurt consumption might be correlated with a broader dietary pattern that includes a higher intake of processed or sugary foods, and even alcohol. Furthermore, while cheese contains unsaturated fatty acids that could be beneficial, it may also be part of an overall dietary pattern that, when combined with other factors, contributes to the observed risk. Therefore, yogurt consumption may be acting as a proxy for these unmeasured or inadequately controlled dietary patterns. In addition, changes in the composition of the gut microbiota should also be considered as a potential explanatory mechanism for these associations.

This study has several notable strengths. First, it focuses on an understudied population, older adults in Iran, thereby filling an essential gap in the global literature on diet and diabetes. Second, rather than examining total dairy intake alone, we separated major dairy subtypes and found distinct associations for yogurt and cheese, which adds nuance to existing evidence. Third, the analysis explicitly considered the cultural and dietary context of Iran, including the widespread consumption of salted doogh or cheese with refined bread, which provides insights that are both realistic and policy‐relevant. Additionally, the use of comprehensive statistical models that adjust for a wide range of demographic, anthropometric and lifestyle covariates strengthens the validity of the results. Finally, by contrasting our findings with those of Western cohorts, this study highlights novel divergences that underscore the importance of regional nutrition research.

Nevertheless, some limitations must be acknowledged. The cross‐sectional design prevents us from drawing causal inferences, as reverse causation cannot be ruled out. Dietary intake was assessed by self‐report, which is subject to recall bias and potential misclassification. Our dietary data did not distinguish between full‐fat and low‐fat milk, sweetened and plain yogurt, or different types of cheese, which limited the precision of our conclusions. The study sample, drawn from Birjand in eastern Iran, may not be fully representative of other Iranian or Middle Eastern populations with differing dietary habits. Finally, despite careful adjustment for multiple confounders, residual confounding from unmeasured factors, such as overall diet quality, genetic predispositions, or differences in gut microbiome, cannot be excluded.

## 5 Conclusions

The findings of this study suggest a nuanced and context‐dependent relationship between dairy consumption and the risk of T2DM in older adults. Although total dairy intake was nearly significantly associated with diabetes prevalence, specific dairy products commonly consumed in Iranian dietary patterns, such as yogurt and cheese, were linked to an increased risk. This observation contrasts with findings from other populations. These results underscore the importance of distinguishing between different types of dairy, as well as considering their quality and processing methods, when evaluating their metabolic effects. Therefore, public health recommendations should move beyond broad generalisations and be tailored to reflect local dietary practices and cultural contexts. To strengthen the evidence base, future research should prioritise well‐designed, longitudinal studies that account for these contextual factors, enabling the development of nutritional guidelines that are both culturally relevant and clinically effective for aging populations.

## Author Contributions

All authors have read and approved the final version of the manuscript, and have contributed significantly to the work, following the recommendations of the International Committee of Medical Journal Editors (ICMJE). Their contributions are detailed below: Conceptualization: Farshad Sharifi, Mehdi Varmaghani and Mitra Moudi. Methodology: Farshad Sharifi and Masoumeh Khorashadizadeh. Software: Mehraneh Movahedi Aliabadi. Data curation: Amirmohammad Tajik, Zeinab Shakeri, Maryam Mohammadi, Hossein Fakhrzadeh, Mitra Moudi and Masoumeh Khorashadizadeh. Investigation: Farshad Sharifi. Formal analysis: Mehdi Varmaghani, Farshad Sharifi, Mahboubeh Darabi and Mitra Moudi. Supervision: Mehdi Varmaghani and Hossein Fakhrzadeh. Funding acquisition: Farshad Sharifi. Project administration: Farshad Sharifi. Writing – original draft: Mahboubeh Darabi, Zeinab Shakeri, Amirmohammad Tajik and Mehdi Varmaghani. Writing – review and editing: Mehdi Varmaghani, Amirmohammad Tajik and Mehraneh Movahedi Aliabadi.

## Funding

The authors have nothing to report.

## Ethics Statement

Ethical approval was obtained from the Endocrinology and Metabolism Research Institute of Tehran University of Medical Sciences (Approval ID: IR.TUMS.EMRI.REC.1396.00158) and the ethical committee of Birjand University of Medical Sciences (Approval ID: IR.BUMS.REC.1397.282).

## Consent

Written informed consent was obtained from all participants prior to enrollment. Participants were informed about the study aims, procedures, and their right to withdraw at any time, and confidentiality was strictly maintained.

## Conflicts of Interest

The authors declare no conflicts of interest.

## Data Availability

The data that support the findings of this study are available from the corresponding author upon reasonable request.
